# CT-based deep learning radiogenomics for predicting key glioma genotypes (IDH, ATRX, EGFR, TP53)

**DOI:** 10.1007/s00234-026-04043-3

**Published:** 2026-06-04

**Authors:** Zohal Alnour Ahmed Emam, Emel Ada, Burçin Pehlivanoğlu, Berrin Çavuşoğlu, Kadir Akgüngör, M. Alper Selver

**Affiliations:** 1 Department of Medical Physics, Dokuz Eylul University, İzmir, Turkey; 2 Department of Radiology, Dokuz Eylül University Hospital, İzmir, Turkey; 3 Department of Pathology, Dokuz Eylül University Hospital, İzmir, Turkey; 4 Department of Atomic and Molecular Physics, Dokuz Eylul University, İzmir, Turkey; 5 Department of Electrical and Electronics Engineering, Dokuz Eylul University, İzmir, Turkey; 6 Ataturk Vocational School of Health Services, Afyonkarahisar Health Sciences University, Afyon, Turkey

**Keywords:** Glioma, Radiogenomics, Computed Tomography, Deep Learning, Molecular Biomarkers

## Abstract

**Purpose:**

Molecular subtyping guides diagnosis and targeted therapy for gliomas. Although MRI—the current imaging standard—can be time-consuming and is sometimes contraindicated, computed tomography (CT) is faster, more widely available, and often preferable in emergency and resource-limited settings. We evaluated whether CT-based radiogenomic signatures combined with machine learning could accurately predict clinically relevant glioma molecular markers.

**Methods:**

In this retrospective study, we characterised non-contrast CT (NCCT) scans from 197 adults with histologically confirmed gliomas. Models were developed to predict mutations in four molecular markers: ATRX (*n*=81), EGFR (*n*=17), TP53 (*n*=71), and IDH (*n*=183). We extracted 208 quantitative radiomic features and added basic demographic variables. Feature selection used LASSO-RFE and Gradient Boosting–RFE with cross-validation. Six classical machine-learning classifiers and deep-learning approaches — including custom fully connected neural networks (FCNN) and TabNet — were trained and compared using ROC–AUC as the primary performance metric.

**Results:**

Deep-learning methods outperformed conventional classifiers for all targets. TabNet achieved ROC–AUCs of 0.900 (95% CI: 0.717–0.989; ATRX), 0.955 (95% CI: 0.661–0.978; TP53), and 0.917 (95% CI: 0.858–0.975; EGFR). A custom FCNN obtained a ROC–AUC of 0.971 (95% CI: 0.876–0.995) for IDH. Cross-validation coefficients of variation were 2.0% for ATRX and 3.0% for TP53 and EGFR, and 16.0% for IDH. Deep-learning approaches yielded statistically significant improvements over conventional methods (*p*-values ranging from <0.05 to <0.001).

**Conclusion:**

NCCT-based analytical methods were able to predict clinically relevant genetic mutations in gliomas and demonstrated performance comparable to established techniques. These findings suggest that CT may serve as a practical option for molecular profiling in urgent or resource-limited settings. Nonetheless, external validation is necessary prior to clinical translation. The EGFR findings, arising from a small EGFR-tested subgroup (n = 17), remain preliminary; the observed performance may not reflect generalisable accuracy, and independent validation in larger cohorts is required.

**Supplementary Information:**

The online version contains supplementary material available at https://doi.org/10.1007/s00234-026-04043-3.

## Introduction

Diffuse gliomas constitute a biologically and clinically heterogeneous group of primary central nervous system tumors, with substantial variation in histopathology, molecular alterations, therapeutic response, and prognosis. They account for approximately 26.3% of primary brain and central nervous system neoplasms, whereas glioblastoma, grade 4, represents 14.2% of cases [1, 2]. This biological variability complicates clinical care: tumors that look the same on routine scans can have very different molecular profiles, and those differences strongly influence prognosis and treatment decisions. In 2016, the World Health Organisation (WHO) introduced an integrated diagnostic approach that required key molecular markers—particularly IDH mutation status and 1p/19q codeletion—for classifying diffuse gliomas, marking a shift toward biologically defined tumor entities. The 2021 WHO update extended that shift by adding further molecular criteria, moving the field away from purely morphology-based labels toward a unified histomolecular classification [1].

Several molecular alterations now carry outsized diagnostic and prognostic importance. IDH mutations are strongly prognostic and alter tumor metabolism in ways that meaningfully change patient outcomes and treatment considerations [3, 4]. Tumor protein p53 (TP53) mutations, which commonly lead to aberrant p53 function, disrupt cell-cycle checkpoints and genomic stability and are linked to distinct tumor behaviors [5]. EGFR undergoes frequent activating mutations and variant-driven structural alterations—most prominently the EGFRvIII mutant form—which enhance proliferative signaling and establish EGFR mutation status as a central molecular feature of glioblastoma biology [6]. Additionally, EGFR activity can interact with p53 pathways to modify tumor biology [7]. ATRX mutations interfere with the normal incorporation of histone H3.3 at telomeric regions, leading to disrupted chromatin structure and enabling the alternative lengthening of telomeres pathway, a hallmark of telomere dysfunction described in gliomas [8].

In clinical practice, these ATRX alterations are found most often alongside IDH mutations, marking a molecular subgroup of gliomas with recognisably distinct biology and prognosis.

Despite the clear value of molecular profiling, routine implementation is constrained by the need for tissue sampling. Obtaining diagnostic material requires stereotactic biopsy or resection, procedures that are not risk-free; hemorrhage, infection, and new neurological deficits are known complications [9]. In addition, intratumoral heterogeneity means that a single biopsy may not capture the full molecular landscape, leaving clinicians with potentially incomplete information for treatment planning [10]. These limitations motivate the search for reliable, noninvasive methods that can augment or, in some settings, substitute for tissue-based assays.

Radiogenomics — the mapping of quantitative imaging features to molecular and genomic traits — offers a promising route to noninvasive molecular characterisation. When paired with machine learning and, increasingly, deep learning approaches, neuroimaging can reveal subtle phenotypes that reflect underlying biology. Most work to date has focused on MRI, where algorithmic models have shown impressive ability to infer IDH status, ATRX loss, and other alterations from imaging alone [11, 12]. Several studies have shown that MRI-derived imaging features can predict molecular markers such as p53, and reviews indicate that these non-invasive imaging approaches hold promise for improving clinical diagnosis and predicting patient outcomes [13]. Reported metrics include pooled sensitivities around 0.831 for IDH, ATRX sensitivities in the 0.682–0.79 range, specificities generally above 0.82, and ROC–AUC values in some series as high as 0.948 for IDH and 0.917 for EGFR [14–17].

Nonetheless, MRI is not universally practical: its high acquisition cost, limited availability in many regions, and contraindications in patients with certain implanted devices restrict routine use in some clinical contexts [18]. In these situations, non-contrast CT (NCCT) frequently serves as the first-line modality because it is rapid, widely available, and effective at revealing mass effect, hemorrhage, bony involvement and other acute findings important for immediate management. NCCT is also amenable to quantitative image analysis and radiomic workflows. Importantly, emerging evidence signifies that NCCT may contain genotype-relevant information: for example, Musigmann et al. (2024) reported IDH prediction from native preoperative CT with an AUC of 0.847 in a 92-patient cohort [19]. Comparable CT-only investigations of ATRX, EGFR, or TP53 are, however, sparse, highlighting a clear need for further, rigorously validated work.

Persistent inequities in access to molecular diagnostics necessitate pragmatic, imaging-based approaches that do not depend on centralised laboratories or high-cost scanners [20–22]. To this end, we developed and evaluated CT-based radiomic signatures together with a suite of machine-learning pipelines — including conventional classifiers, custom fully connected neural network (FCNN) [23, 24], and TabNet— to assess whether features extractable from preoperative NCCT convey clinically actionable molecular information [25]. We targeted four molecular alterations—IDH, ATRX, EGFR and TP53—because each has well-established implications for glioma classification, prognosis and therapeutic response; accurate, noninvasive estimates of these markers could therefore refine preoperative risk stratification and influence both surgical planning and adjuvant treatment decisions. To ensure that any observed signal was robust, our NCCT pipeline combined standardised image preprocessing and careful segmentation with principled feature selection and rigorous model validation. Specifically, we applied stability-aware feature filtering together with regularised selection methods, trained a range of classifiers (conventional algorithms, FCNN and TabNet) within a nested cross-validation framework, and evaluated final models on held-out data where available to reduce overfitting and enable fair comparison with MRI-based approaches. By quantifying CT’s predictive performance under these controlled conditions, we aimed to clarify the practical, translational value of a widely available imaging modality in settings where MRI or all-Inclusive molecular testing are unavailable or delayed.

## Materials and methods

**Fig. 1:**
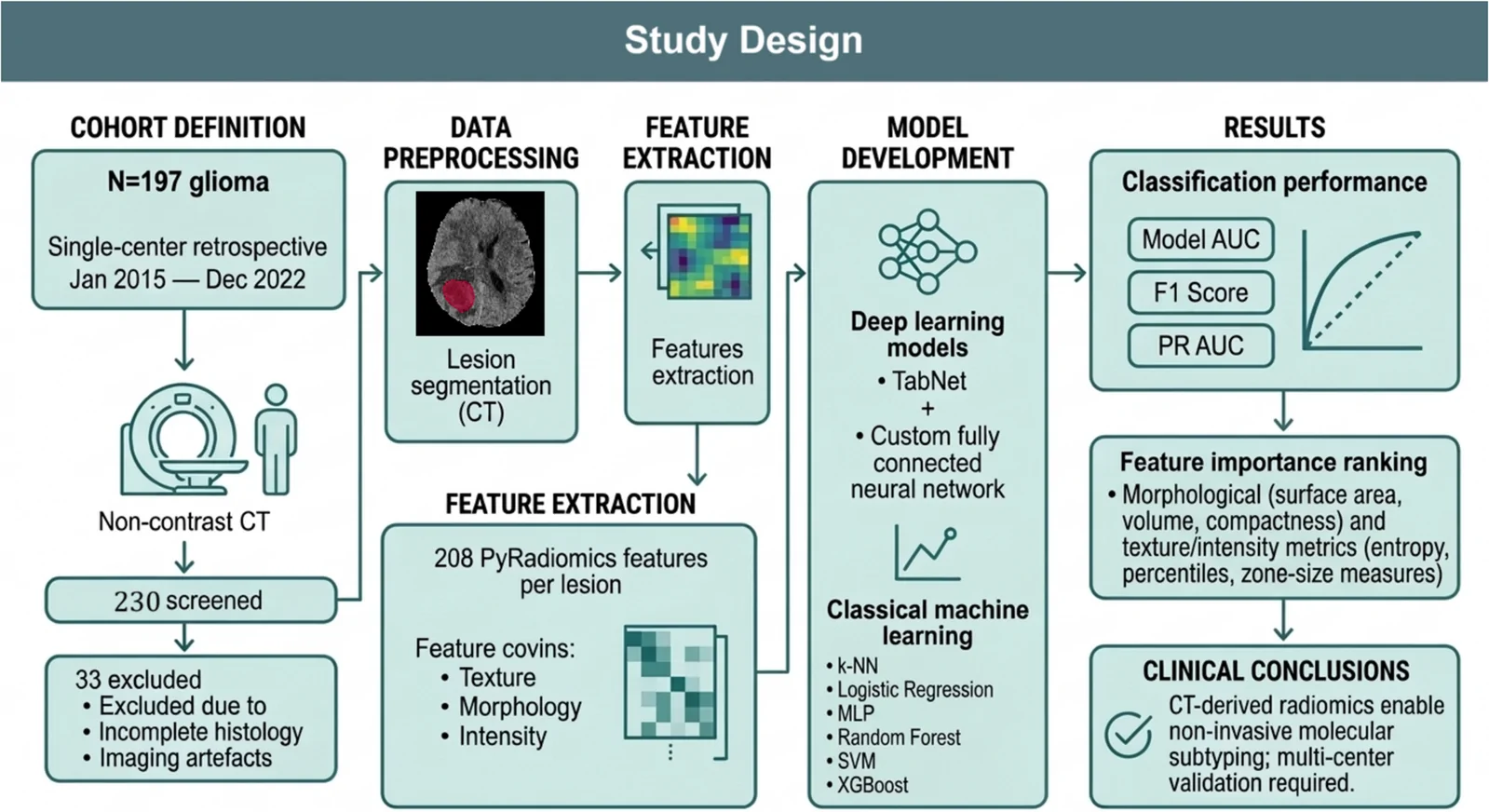
Study design and radiomics workflow for CT-based molecular subtyping of glioma, from cohort selection and lesion segmentation to feature extraction, model development, performance evaluation, and clinical interpretation

This study developed a CT-centred radiogenomics pipeline to predict molecular biomarkers in gliomas, with model development and reporting guided by the TRIPOD-AI framework for clinical prediction models that incorporate artificial intelligence [26]. The overall study design and radiomics-based analytical workflow are illustrated in Fig. 1.

### Study cohort and molecular data

Non-contrast head CT examinations performed between January 2015 and December 2022 were retrospectively reviewed. Of 230 available studies, 197 adult patients with histopathologically confirmed gliomas met the inclusion criteria and were retained for analysis [27]. The cohort was dominated by glioblastoma (154 cases, 78.2%), followed by anaplastic astrocytoma (17 cases, 8.6%), diffuse astrocytoma (11 cases, 5.6%), anaplastic oligodendroglioma (10 cases, 5.1%), and oligodendroglioma (5 cases, 2.5%). Although the original pathology reports followed pre-2021 criteria, all tumours were subsequently reclassified according to the World Health Organization Classification of Tumours of the Central Nervous System, fifth edition (WHO CNS5), as shown in Fig. S1. Reclassification yielded glioblastoma, IDH-wildtype (153/197; 77.7%), astrocytoma, IDH-mutant (22/197; 11.2%), and oligodendroglioma, IDH-mutant and 1p/19q-codeleted (13/197; 6.6%); the remaining 9 cases (4.6%) lacked sufficient molecular information for definitive WHO CNS5 assignment.

**Fig. 2:**
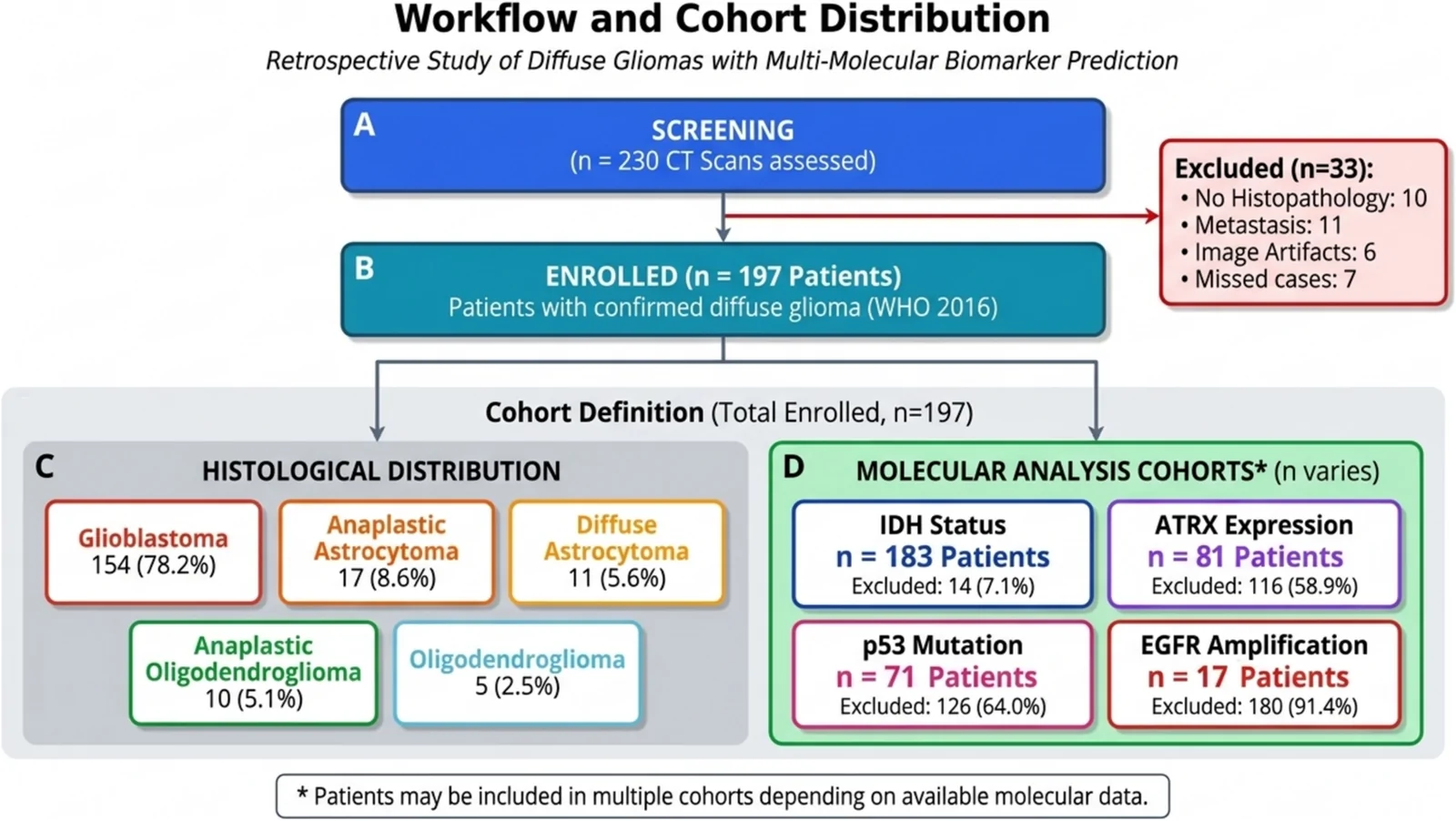
Cohort selection workflow and distribution of histological and molecular subgroups. (**A**) Initial screening of CT scans (*n* = 230) and exclusion criteria. (**B**) Final enrolled cohort of patients with confirmed diffuse glioma (*n* = 197). (**C**) Histological distribution of glioma subtypes within the cohort. (**D**) Molecular analysis cohorts for IDH status, ATRX expression, TP53 mutation, and EGFR amplification, with corresponding sample sizes and exclusions

Biomarker-specific datasets were subsequently constructed according to the availability of genotype data, resulting in cohort sizes of ATRX (*n* = 81), EGFR (*n* = 17), TP53 (*n* = 71), and IDH (*n* = 183). Molecular testing was performed for IDH in 183 cases (93%), ATRX in 81 (41%), TP53 in 71 (36%), and EGFR in 17 (9%). Patients were permitted to contribute to more than one biomarker cohort where corresponding molecular assays were available. Figure 2 summarises the screening process, exclusion of ineligible cases, and final allocation to molecular subgroups.

### Image acquisition and preprocessing

All scans were acquired using a standardised protocol, with a tube voltage of 120 kVp and tube current ranging from 165 to 300 mAs. Native slice thickness varied between 0.5 and 3 mm. Examinations were performed on 16- and 64-slice scanners (Philips Healthcare) and a 160-slice scanner (Toshiba Medical Systems) throughout the study period, without major protocol changes expected to affect radiomic feature reproducibility. Minor inter-year variation in mAs reflected routine clinical dose modulation. To mitigate the influence of scanner- and acquisition-related variability, skull stripping was applied, image volumes were resampled to isotropic spacing, and intensity discretisation and spatial normalisation were applied in accordance with Image Biomarker Standardisation Initiative (IBSI) recommendations. Image processing and analysis were conducted on anonymised data, following institutional review board approval with a waiver of informed consent for retrospective research use.

### Tumor segmentation and radiomic feature extraction

Tumor segmentation used a semi-automated approach implemented in the Medical Imaging Interaction Toolkit (MITK, version 2021.10) [28]. An experienced neuroradiologist generated the initial contours, with manual refinement applied where necessary, and a final quality-control review was conducted by the same reader to ensure consistency and accuracy of the delineations. Annotation focused on the tumour core, defined as the solid central tumour component visible on non-contrast CT, and MRI was used in certain cases, where patient data were available, to confirm boundary delineation. In high-grade gliomas, this region typically appears heterogeneous and may include necrotic or haemorrhagic elements, whereas in low-grade gliomas it is usually more homogeneous and subtly hypo- or iso-dense. Peritumoural oedema was excluded from segmentation to avoid inclusion of non-neoplastic tissue and to preserve the tumour specificity of the radiomic features.

From each final region of interest, we extracted a set of quantitative descriptors using the Medical Imaging Toolbox in MATLAB (The MathWorks, Inc., 2024) [29]. The feature set comprised 208 radiomic features plus two clinical covariates (age and gender), yielding a total of 210 variables for downstream modelling. Feature types included: morphological metrics (*n* = 22) capturing three-dimensional geometry (volume, surface area, compactness, sphericity and related measures); first-order intensity statistics (*n* = 30) describing central tendency, dispersion and higher-order moments computed on both original and discretised intensities; histogram-derived descriptors (*n* = 12) summarising voxel-value distributions and gradient relationships; and an extensive texture panel (*n* = 144) calculated from six established texture matrices — Gray Level Co-occurrence Matrix (GLCM, 50 features aggregated across orientations), Gray Level Run Length Matrix (GLRLM, 32 features), Gray Level Size Zone Matrix (GLSZM, 16 features), Gray Level Distance Zone Matrix (GLDZM, 16 features), Neighborhood Gray Tone Difference Matrix (NGTDM, 5 features) and Gray Level Dependence Matrix (GLDM, 17 features) — together with eight additional texture descriptors.

Preprocessing for radiomic analysis followed a standardised sequence to promote stability and comparability. Native CT volumes underwent isotropic resampling. Image intensities were interpolated using a B-spline kernel, whereas region-of-interest masks were resampled using linear interpolation. The resampled voxel spacing was defined as the ceiling of the minimum original voxel dimension to avoid artificial down-sampling.

Intensity discretisation employed a fixed-bin approach, whereby the number of bins for each volume was automatically computed as the intensity range multiplied by 0.1 and rounded to the nearest integer. Resegmentation was used to exclude voxels outside anatomically relevant intensity ranges and to remove extreme outliers that could otherwise affect texture feature calculation. All feature extraction procedures followed IBSI recommendations to support reproducibility and comparability across studies.

### Data splitting and feature selection

Stratified random sampling partitioned the cohort with target-specific ratios to address limited sample sizes and uneven mutation prevalences. For IDH and TP53 we used an 80%/20% train/test split with 20% of the training set reserved for validation. ATRX and EGFR experiments used a 60%/40% train/test split with the same internal validation fraction. Class imbalance was handled only on training folds using a hybrid resampling pipeline combining SMOTE [30], ADASYN [31], and Tomek Links removal [32] to avoid contaminating held-out data.

For EGFR specifically, the tested subset was small (*n* = 17; 8.6% of the full cohort), with 10 positive cases (58.8%) and 7 negative cases (41.2%). Given this pronounced imbalance, resampling was applied with particular care within the training folds only.

Radiomic features were standardised using z-score normalisation fitted on the training data and applied unchanged to the validation and test sets. To prevent information leakage, all feature selection steps, including LASSO regularisation and recursive feature elimination with cross-validation (RFECV) using a Gradient Boosting classifier as the estimator, were performed exclusively within training folds during cross-validation. Feature selection was repeated independently for each fold, and no information from validation or held-out test sets was used to select or rank features at any stage. This nested design helps ensure that reported performance estimates are not inflated by artefacts of data reuse.

Feature selection was adapted by model family. TabNet pipelines applied a hybrid LASSO followed by Recursive Feature Elimination (RFE) [33, 34]: LASSO regularisation (alpha = 0.001) first discarded features with zero coefficients, RFE then ranked features and selected the top 20, and the final TabNet set was defined as the intersection between LASSO-selected and RFE-selected features to retain consistently important predictors. FCNN and traditional machine-learning pipelines adopted a Gradient Boosting Classifier–based RFECV selection with the gradient boosting estimator as base, five-fold cross-validation and accuracy scoring to evaluate candidate subsets.

### Model development and training

The comparative structure evaluated six conventional classifiers and two deep architectures. Conventional machine learning models included k-Nearest Neighbors, Logistic Regression, Multilayer Perceptron, Random Forest, Support Vector Machine, and Extreme Gradient Boosting, with hyperparameter tuning performed across standard model-specific parameter spaces as detailed in Table S1.

**Fig. 3:**
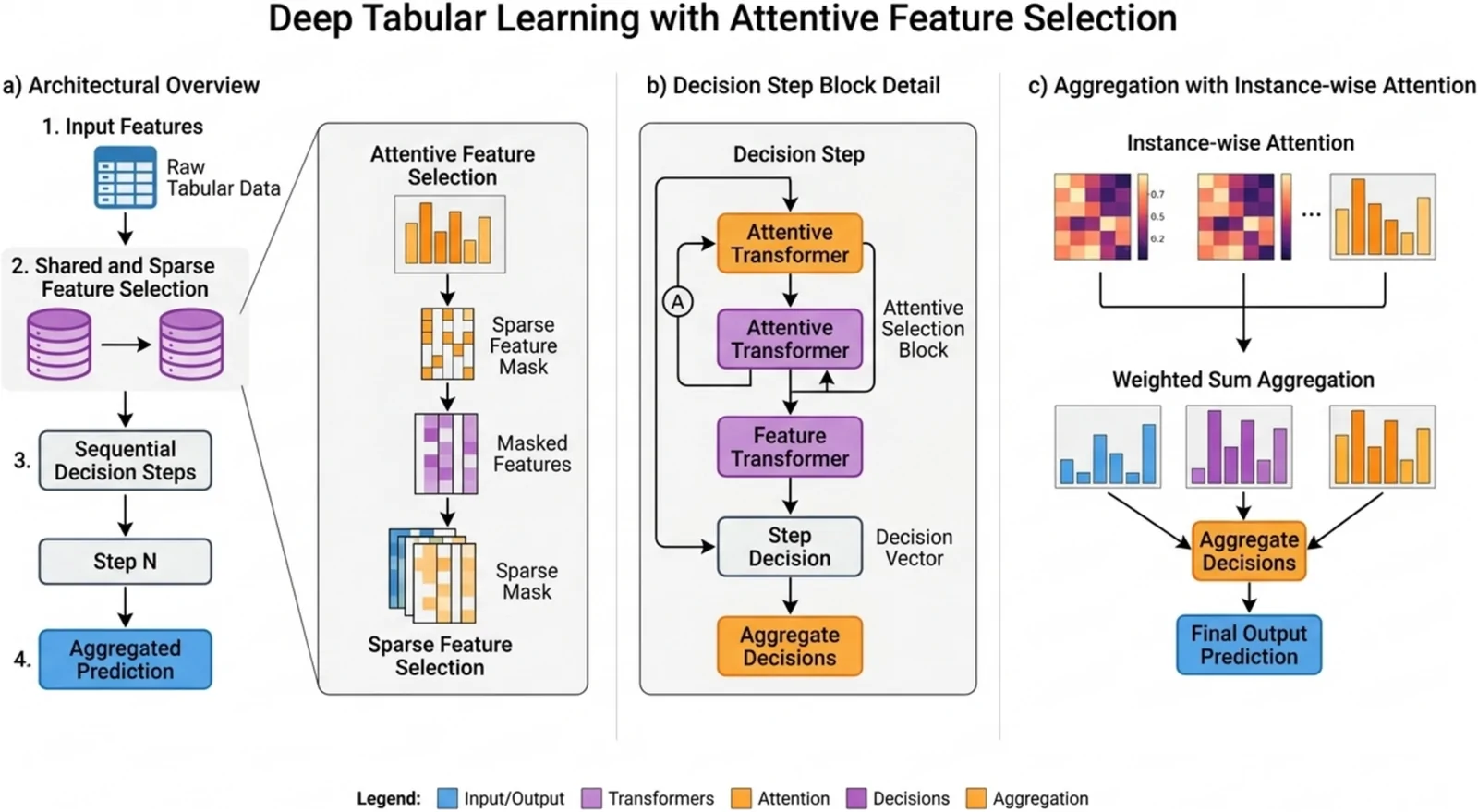
Schematic representation of the proposed deep tabular learning framework. (**a**) Raw tabular inputs are processed through a shared sparse selection mechanism that progressively refines feature usage across sequential decision steps, culminating in a unified prediction. (**b**) Each decision step employs an attentive selection block, where feature relevance is dynamically modulated via transformer-based representations to produce a step-specific decision vector. (**c**) Step-wise outputs are combined through instance-wise attention using a weighted aggregation scheme, yielding the final prediction. Color coding denotes the functional components, including data flow, transformation modules, attention mechanisms, decision units, and aggregation

**Fig. 4:**
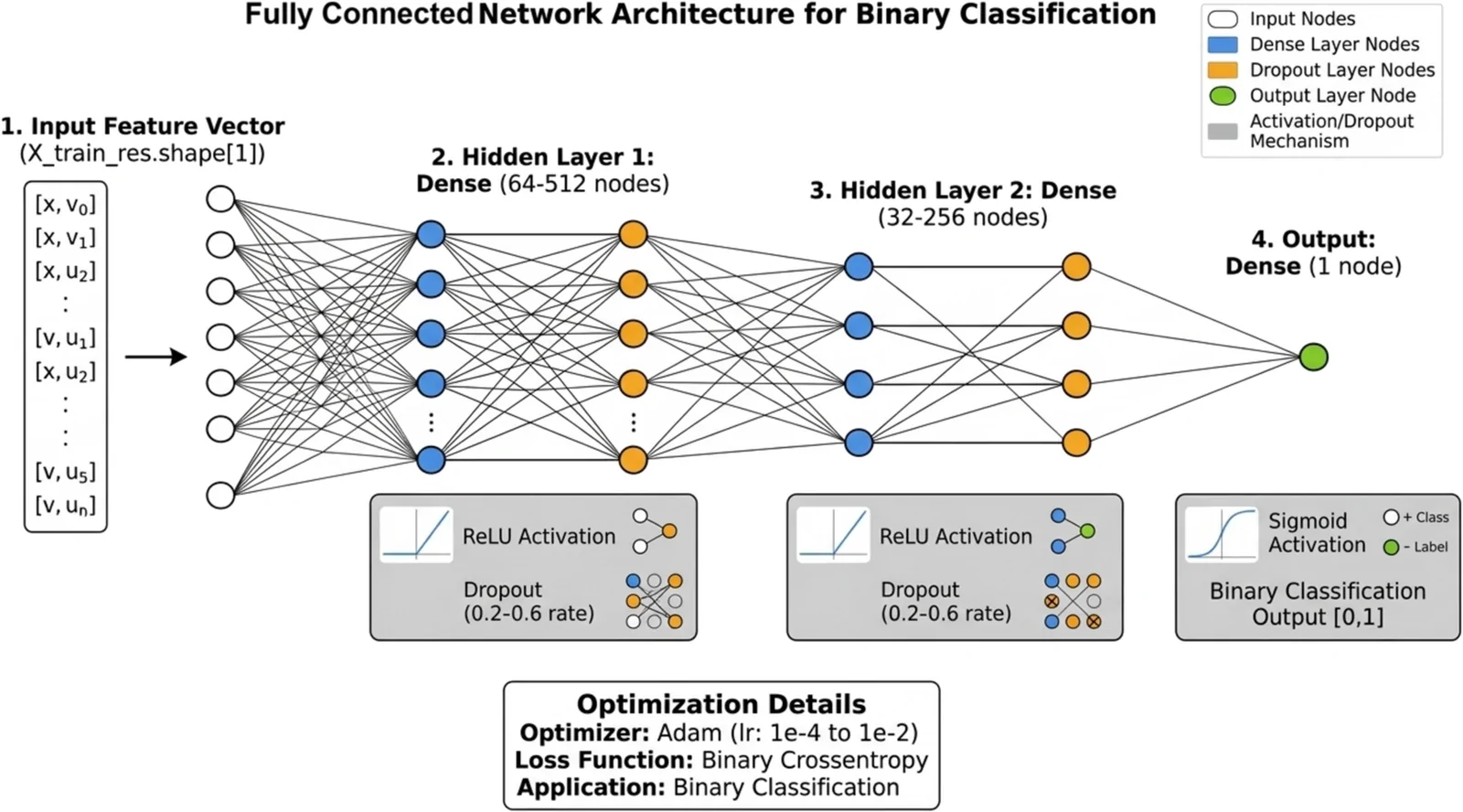
Architecture of the fully connected neural network designed for two-class classification. The model receives an input feature vector and propagates it through two dense hidden layers with progressively reduced dimensionality, enabling hierarchical feature abstraction. Each hidden layer incorporates ReLU activation and dropout regularization to enhance nonlinearity and mitigate overfitting. The final output layer consists of a single neuron with sigmoid activation, producing a probability score in the range [0,1]. Model optimization is performed using the Adam optimizer with cross-entropy loss, reflecting its suitability for probabilistic outcome prediction

Deep architectures included TabNet, an interpretable attention-based model for tabular data (Fig. 3), and a custom FCNN, architecturally distinct from convolutional neural networks (CNNs) which process spatial image grids, with dropout regularisation (Fig. 4). The schematic shows TabNet’s sequential attention across multiple decision steps (n_steps = 3–10). At every step, the model applies a transformer-style block made of a decision module (8–64 units) and an attention module (8–64 units). The network selects features implicitly through sparse attention masks (Sparsemax); we tuned the sparsity parameter γ between 1.0 and 2.0 and the attention regulariser λ_sparse between 1e-4 and 1e-3. By alternately selecting informative inputs and transforming them, the network builds hierarchical representations and aggregates step-wise outputs into a final binary prediction. The FCNN architecture consisted of two fully connected hidden layers (64–512 and 32–256 units) with ReLU activation, dropout regularisation (0.2–0.6), and a sigmoid output layer for binary classification. Training used the Adam optimiser (learning rate range: 1e − 4–1e − 2) with binary cross-entropy loss. Hyperparameter tuning was performed via Bayesian search in Optuna, based on the Tree-structured Parzen Estimator framework [35, 36].

To mitigate overfitting and optimistic selection bias we used nested cross-validation [37], architecture regularisation and early stopping. Missing radiomic values were excluded at extraction then mean-imputed within training and validation folds to prevent leakage [38]. Feature standardisation and correlation-based filtering with a 0.40 threshold reduced redundancy [39, 40]. Internal validation was performed using stratified five- to three-fold cross-validation [41]; uncertainty quantification was conducted using bias-corrected and accelerated bootstrap with 1,000 iterations [42]; and model stability and resilience to simulated noise perturbations were evaluated using multi-norm perturbation strategies inspired by adversarial machine-learning approaches [43].

### Statistical analysis

Model performance was evaluated using ROC-AUC, PR-AUC, and F1 score, reported as mean ± SD across validation folds. A composite ranking was derived by averaging the normalised values of these three metrics to facilitate model comparison. Ninety-five per cent confidence intervals (CIs) for ROC-AUC were estimated using the Bias-Corrected and Accelerated (BCa) bootstrap method (B = 2,000 resamples; α = 0.05), applied to point estimates derived from the held-out test set [44, 45]. For the cross-validation experiment, 95% CIs were estimated using the corrected resampled t-statistic to account for statistical dependence between overlapping training folds [46, 47].

Pairwise comparisons of ROC curves were performed using DeLong’s non-parametric test [48, 49, 50]. Feature importance was examined using TabNet’s native attention weights for the TabNet model, mean decrease in impurity from the Gradient Boosting feature selection stage for the fully connected neural network [51], and permutation importance applied to tree-based classifiers [52]. All analyses were implemented in Python (v3.12.0) using standard scientific libraries [53–55]; statistical significance was set at *p* < 0.05. Figures were produced with Matplotlib and Seaborn [56, 57]. Theoretical derivations are provided in Appendix A.

## Results

### Cohort characteristics

**Fig. 5:**
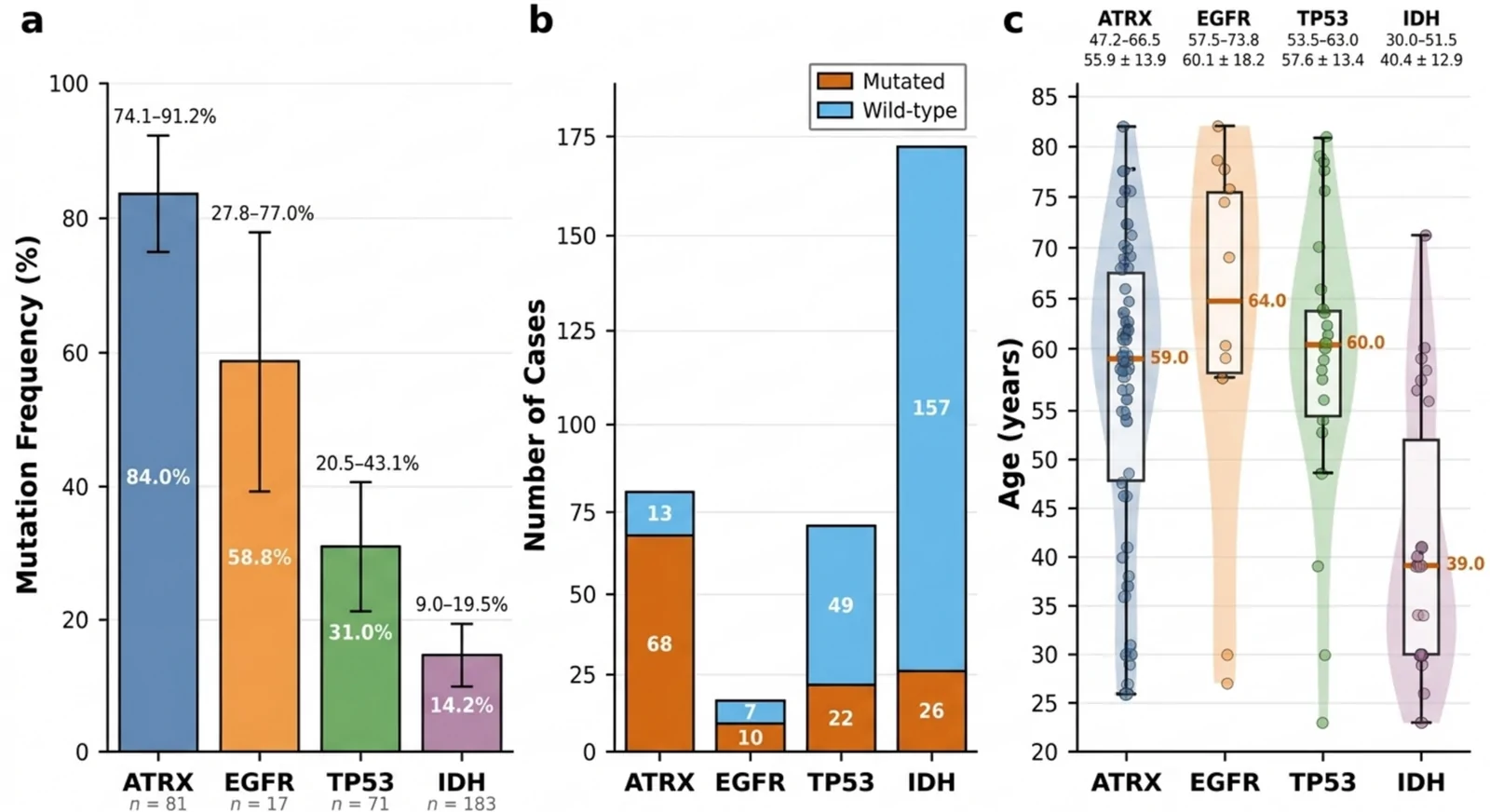
Molecular and demographic characteristics of the study cohort (*N* = 197). (**a**) Pathogenic mutation frequency in key cancer-associated biomarkers across the cohort. In the evaluated samples, ATRX mutations were the most common (84.0%, *n* = 81). EGFR and TP53 mutations were observed in 58.8% (*n* = 17) and 31.0% (*n* = 71) of cases, respectively, whereas IDH mutations occurred in 14.2% (*n* = 183) of cases. Error bars indicate 95% Clopper–Pearson confidence intervals. (**b**) Absolute numbers of mutated versus wild-type cases for each biomarker. ATRX is predominantly mutated (68/81 cases), while TP53 and IDH show a majority wild-type status with 49/71 and 157/183 cases, respectively. EGFR shows fewer wild-type cases (7/17). (**c**) Age distribution among altered and mutation carriers only. EGFR-mutated patients are the oldest (median: 64.0 years, IQR: 57.5–73.8), while IDH-mutated patients are the youngest (median: 39.0 years, IQR: 30.0–51.5.0.5). ATRX and TP53 carriers show intermediate ages (median: 59.0 and 60.0 years, respectively; IQR: 47.2–66.5 and 53.5–63.0, respectively). Violin plots show age distribution density; box plots display median (orange line) and interquartile ranges; individual data points are overlaid

Alteration prevalences among tested cases were 14.2% for IDH mutation, 84.0% for ATRX loss, 31.0% for TP53 mutation, and 58.8% for EGFR amplification. Demographics by biomarker cohort are summarised in Table 1 and depicted in Fig. 5: mean ages ranged from 56.92 to 62.94 years, and male predominance persisted across groups (60.1–82.4%). The IDH-mutant cohort had a mean age of 57.34 ± 13.88 years with 60.1% male representation; ATRX-tested participants averaged 57.06 ± 14.07 years (63.0% male); EGFR-tested participants were oldest (62.94 ± 12.12 years; 82.4% male); and the TP53 cohort averaged 56.92 ± 14.34 years with 62.0% male. Independent t-tests found no significant age differences between sexes within any biomarker cohort (all *p* > 0.05).

**Table 1:** Demographic and clinical characteristics of glioma patients by genetic mutation status (n varies by gene)

Characteristic	ATRX (*n* = 81)	EGFR (*n* = 17)	TP53 (*n* = 71)	IDH (*n* = 183)
Mutation Status				
Mutated	68 (84.0)	10 (58.8)	22 (31.0)	26 (14.2)
Wild type	13 (16.0)	7 (41.2)	49 (69.0)	157 (85.8)
Age (years)ᵃ	57.1 ± 12.5	62.9 ± 10.8	56.9 ± 13.2	51.2 ± 14.7
Median age	59.0	63.0	59.0	53.0
Sex				
Male	51 (63.0)	14 (82.4)	44 (62.0)	108 (59.0)
Female	30 (37.0)	3 (17.6)	27 (38.0)	75 (41.0)

ᵃ Mean ± standard deviation; values presented as *n* (%)

### Predictive algorithm results

Multiple machine learning architectures were trained and compared for predicting glioma-associated genetic alterations using AUC, F1 score, PR AUC, composite score, and rank across ATRX, EGFR, IDH, and TP53 tasks (Table S2). TabNet achieved the highest composite score and best overall discriminative performance for ATRX, EGFR, and TP53 prediction. In contrast, a custom FCNN demonstrated the strongest performance for IDH mutation classification, outperforming the remaining models across the set of evaluation metrics.

Regarding target-specific discrimination metrics, the FCNN achieved an IDH ROC–AUC of 0.971, followed closely by logistic regression at 0.967 and TabNet at 0.890. For ATRX, TabNet reached a ROC–AUC of 0.900 with an F1 score of 0.926, outperforming Random Forest (ROC–AUC = 0.739) and SVM (ROC–AUC = 0.671). EGFR prediction showed TabNet ROC–AUC = 0.917 and F1 = 0.889, versus 0.750 for both SVM and RF. For TP53, TabNet achieved a ROC–AUC of 0.955 and F1 = 0.727, while the FCNN reached a ROC–AUC of 0.659.

**Fig. 6:**
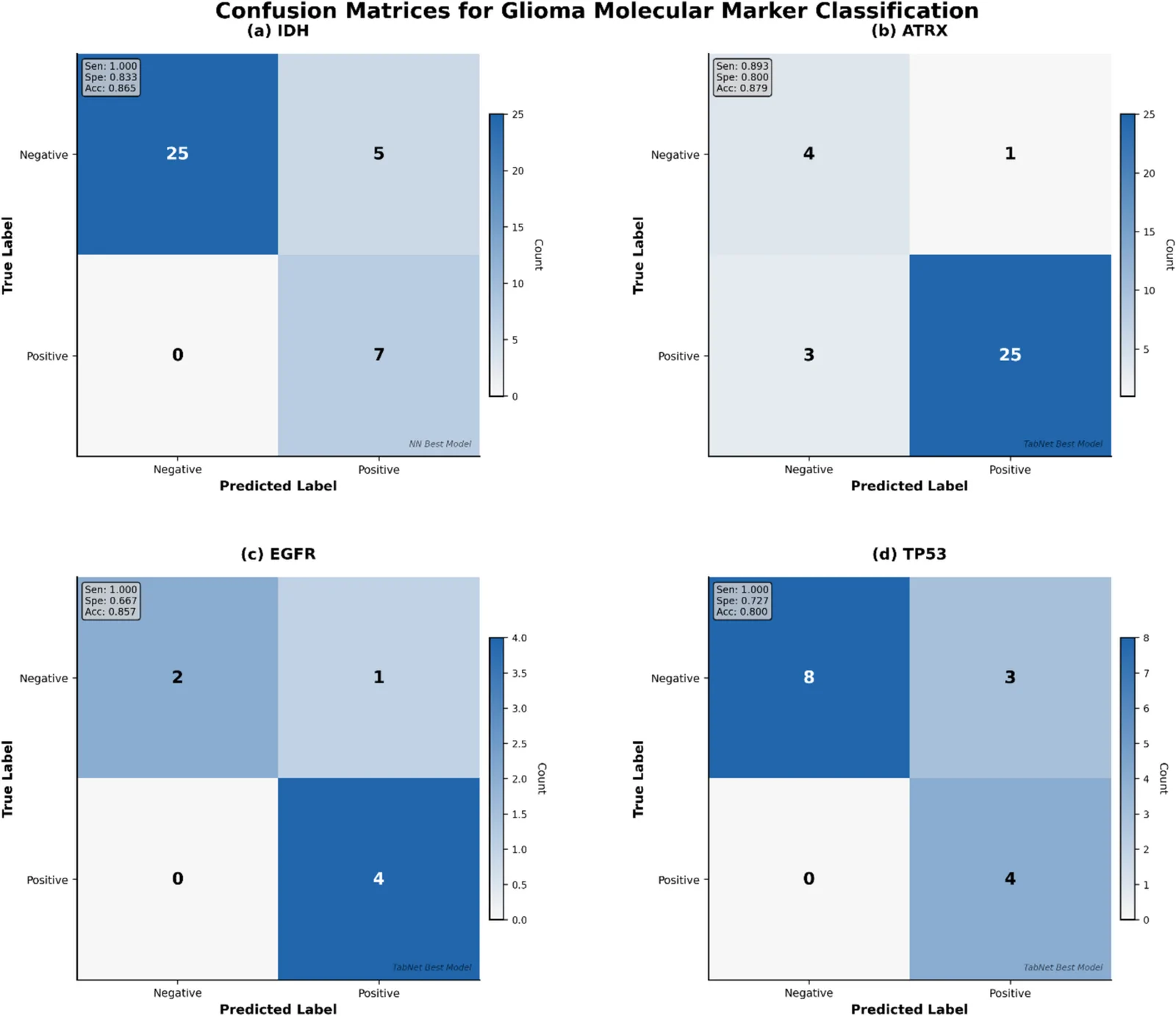
Confusion matrices illustrating classification outcomes for four glioma molecular markers using the best-performing models per target. (**a**) IDH — custom FCNN: sensitivity 1.000, specificity 0.833, accuracy 0.865 (7 true positives, 25 true negatives, 5 false positives, 0 false negatives). (**b**) ATRX — TabNet: sensitivity 0.893, specificity 0.800, accuracy 0.879 (25 true positives, 4 true negatives, 1 false positive, 3 false negatives). (**c**) EGFR — TabNet: sensitivity 1.000, specificity 0.667, accuracy 0.857 (4 true positives, 2 true negatives, 1 false positive, 0 false negatives). (**d**) TP53 — TabNet: sensitivity 1.000, specificity 0.727, accuracy 0.800 (4 true positives, 8 true negatives, 3 false positives, 0 false negatives). Each panel reports absolute counts with corresponding proportions, where color intensity reflects cell magnitude

**Fig. 7:**
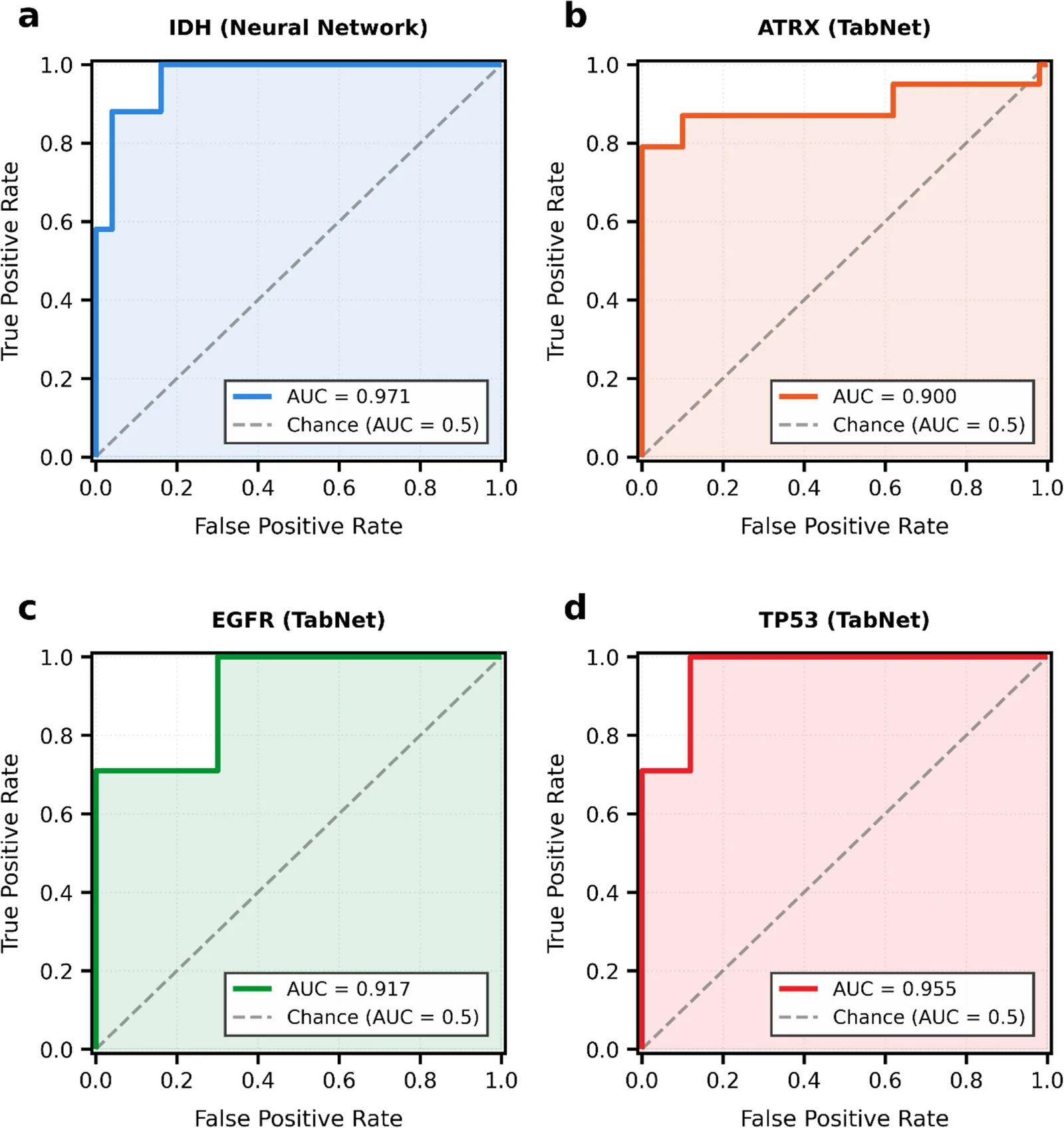
Receiver Operating Characteristic (ROC) curves demonstrating discriminative performance of optimised machine-learning models for genetic target prediction in glioma. Panel (**a**) shows the FCNN model for IDH mutation (AUC = 0.971). Panels (**b**–**d**) display TabNet results for ATRX loss (AUC = 0.900), EGFR amplification (AUC = 0.917) and p53 mutation (AUC = 0.955). Each curve plots true positive rate against false positive rate across thresholds; AUC quantifies overall discrimination while the diagonal line (AUC = 0.5) denotes chance. Models were selected on cross-validation performance and interpretability of radiomic signatures. Variation in AUC values shows that some biomarkers produce stronger, more detectable signals on CT than others, helping to prioritise which markers are suitable for CT-based prediction

**Fig. 8:**
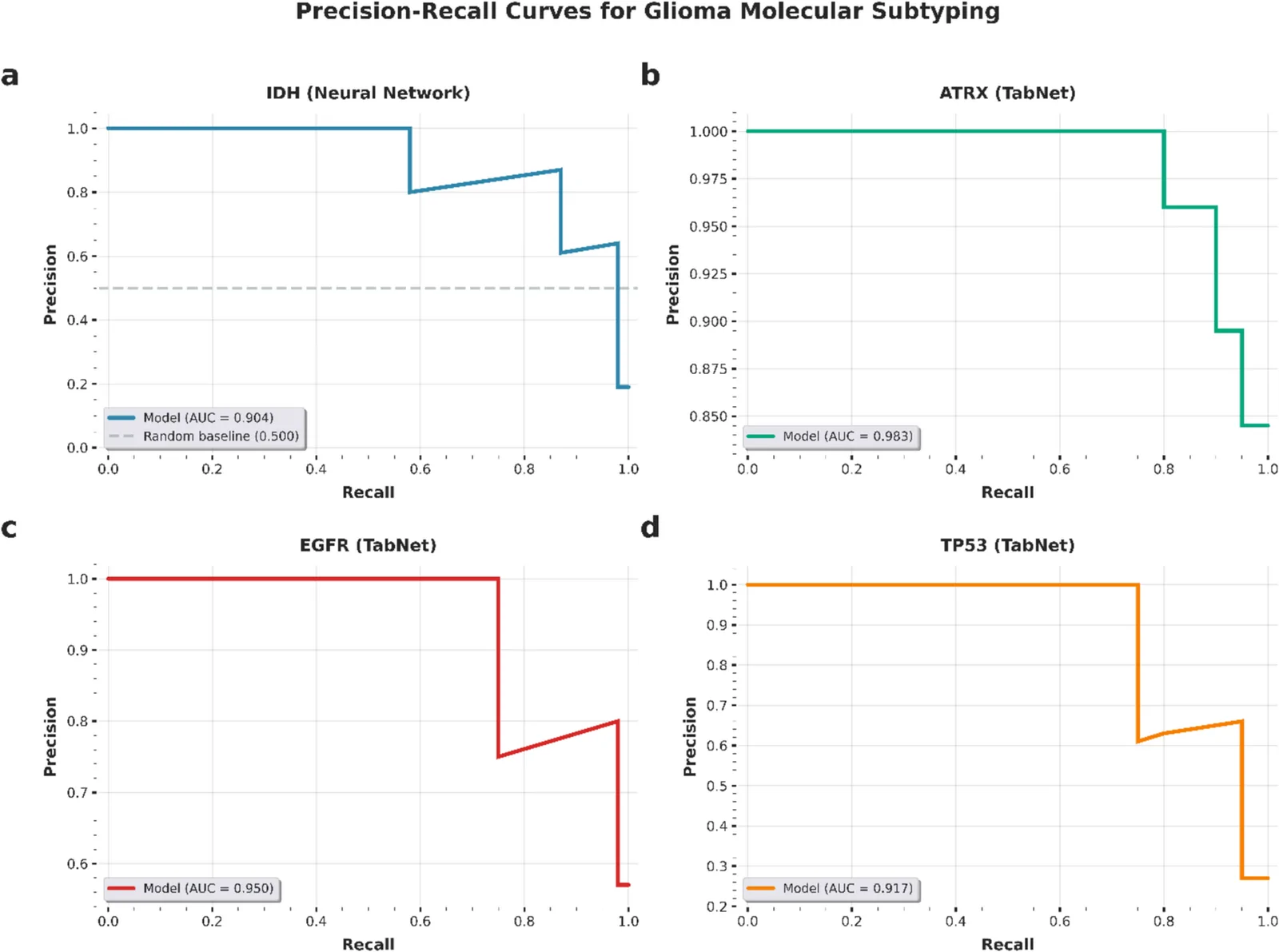
Precision–recall curves illustrate model performance in imbalanced classification settings typical of glioma molecular subtyping. Panel (**a**) shows the FCNN model for IDH mutation (AUPRC = 0.904). Panels (**b**–**d**) present TabNet results: ATRX loss (AUPRC = 0.983), EGFR amplification (AUPRC = 0.950) and TP53 mutation (AUPRC = 0.917). Each curve maps precision (positive predictive value) against recall (sensitivity) across thresholds; the area under the precision–recall curve (AUPRC) better reflects performance when positive classes are scarce. The baseline line indicates expected precision for a random classifier given class prevalence

Case-level confusion summaries included: ATRX loss — 25/28 true positives (89.3%) and 4/5 true negatives (80.0%); TP53 mutation — 4/4 true positives (100.0%) and 8/11 true negatives (72.7%); EGFR amplification — 4/4 true positives (100.0%) and 2/3 true negatives (66.7%); IDH mutation — 7/7 true positives (100.0%) and 25/30 true negatives (83.3%). These classification outcomes are shown in Fig. 6, with separate confusion matrices for each molecular marker. ROC and PR curves are presented in Figs. 7 and 8.

### Validation and feature signatures

**Fig. 9:**
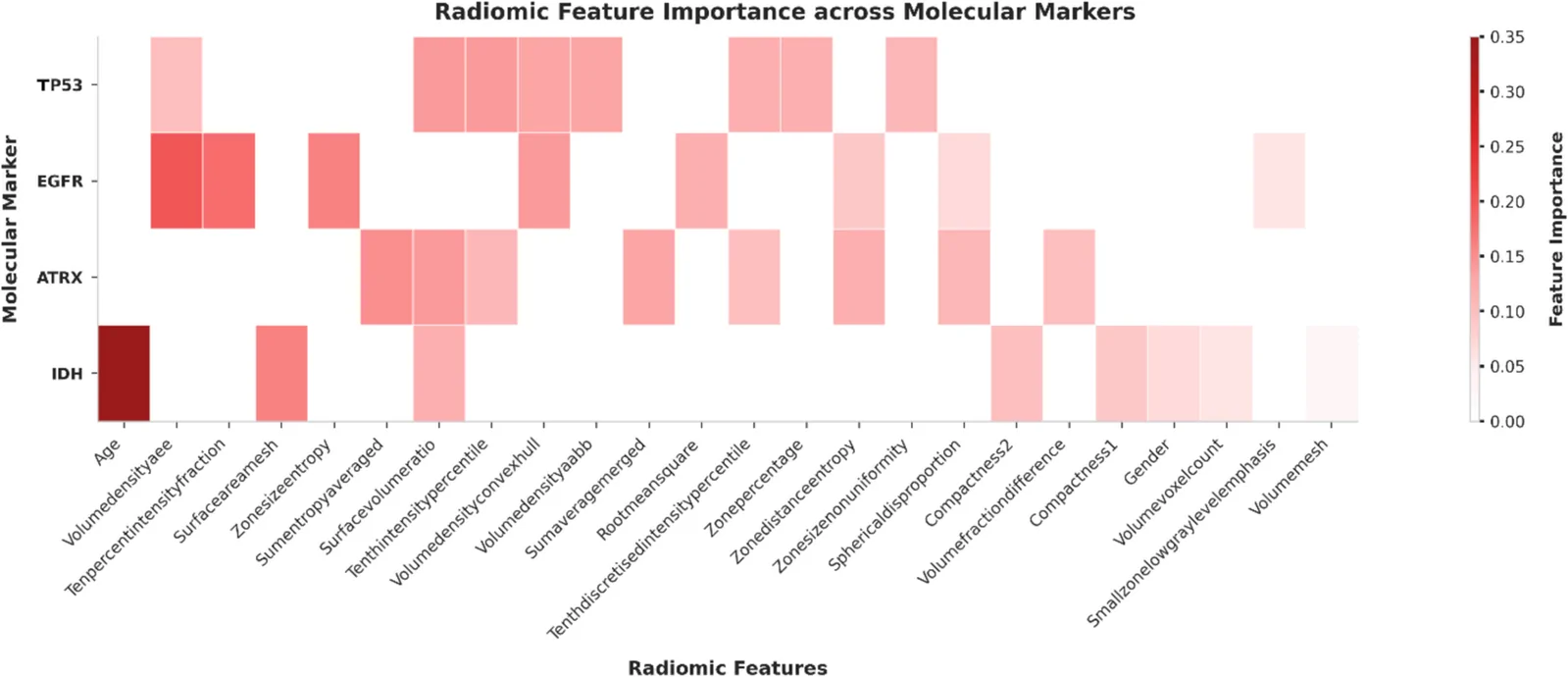
Radiomic feature importance heatmap illustrating the relative contribution of 24 variables to the prediction of four molecular targets in glioma (IDH, ATRX, EGFR, and TP53). Features are organized into demographic (age), morphological (e.g., surface area, volume, compactness), and texture/intensity-derived descriptors (e.g., entropy, intensity percentiles, and zone-size metrics). Color intensity reflects the magnitude of feature importance, with darker shades indicating greater influence on model predictions. Age emerges as the most dominant contributor for IDH, whereas volume-related density measures and fine-grained texture descriptors show higher relevance for EGFR and ATRX, respectively. These target-specific importance patterns improve model interpretability, inform feature prioritization across cross-validation, and delineate the radiomic domains most influential for predictive performance

DeLong testing confirmed statistically significant superiority of top-performing models for each target (Table 2). The IDH classification performed using FCNN significantly outperformed RF (*p* = 0.040), MLP (*p* = 0.018), XGBoost (*p* = 0.012), SVM (*p* = 0.005), and TabNet (*p* = 0.002). TabNet’s advantage for ATRX, TP53 and EGFR was likewise significant (all *p* < 0.05). Feature-importance profiles revealed distinct radiomic signatures (Fig. 9): IDH relied on patient age (0.35) and surface-to-volume ratio (0.18); ATRX on spherical disproportion (0.24) and sum entropy (0.19); TP53 on convex-hull volume density (0.22) and zone-size non-uniformity (0.17); EGFR on Global Intensity Peak 3D (0.34) and Small Zone Low Gray Level Emphasis 3D (0.33).

**Table 2:** DeLong test results for significance of AUC differences between models

Gene	Reference Model	AUC	%95 CI	Comparison Model	*p*-value	Significance
IDH	Fully connected neural network	0.971	0.876–0.995	K-Nearest Neighbors	0.059	NS
				Logistic Regression	0.795	NS
				MLP	0.018	*
				Random Forest	0.040	*
				SVM	0.005	**
				TabNet	0.002	**
				XGBoost	0.012	*
ATRX	TabNet	0.900	0.717–0.989	All other models	< 0.01	**
EGFR	TabNet	0.917	0.858–0.975	Logistic Regression	0.015	*
				MLP	0.015	*
				XGBoost	0.001	**
TP53	TabNet	0.955	0.661–0.978	All other models	< 0.001	***

NS = Not Significant (*p* > 0.05)

* *p* < 0.05; ** *p* < 0.01; *** *p* < 0.001

Reference models represent the top-performing model for each molecular target

**Fig. 10:**
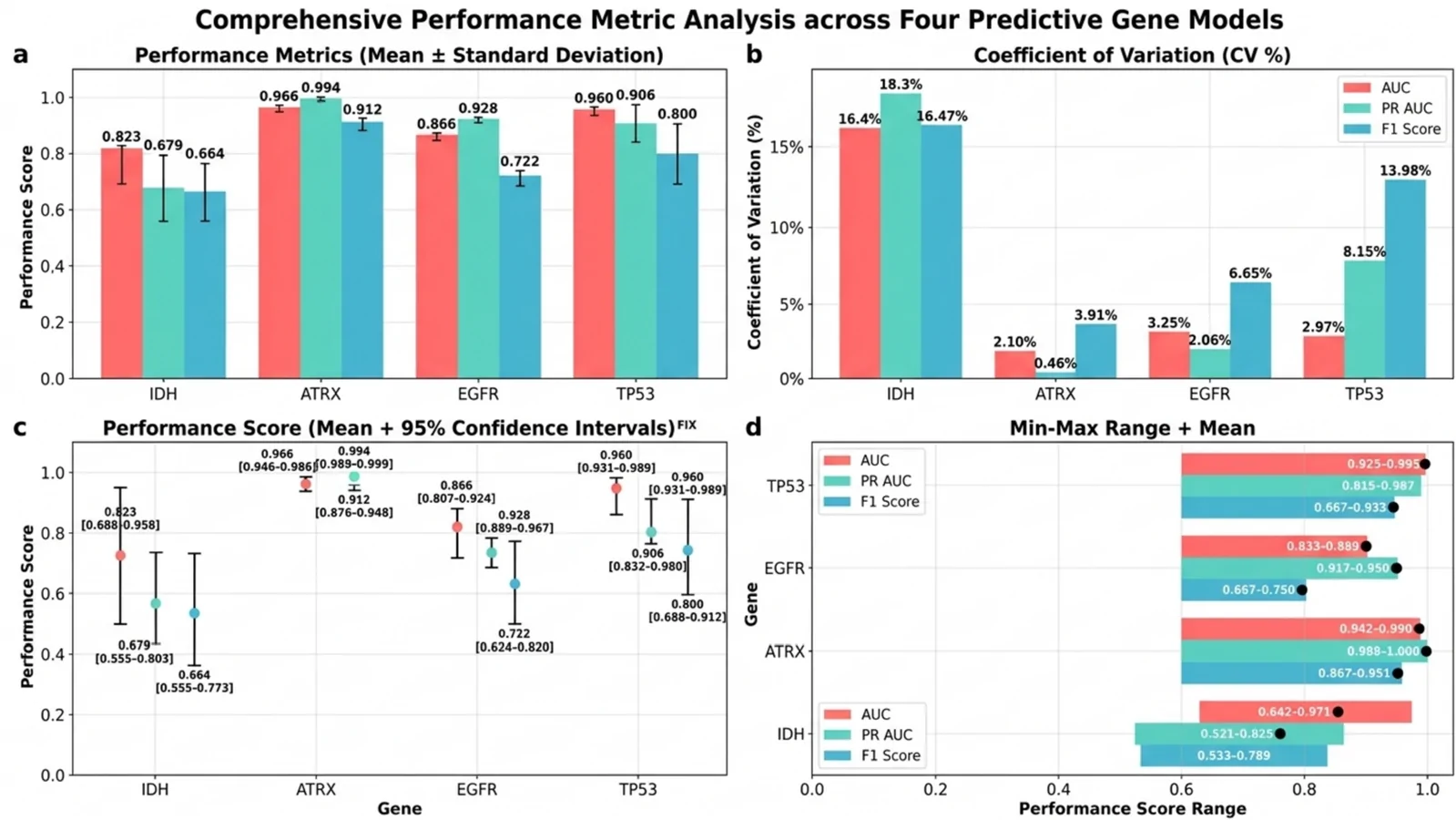
Summary of model performance for genetic biomarker prediction across multiple targets and evaluation metrics. (**a**) Mean AUC, PR–AUC, and F1 scores are presented for each gene, with standard deviation bars and annotated mean values to facilitate direct comparison. (**b**) Coefficient of variation (CV) analysis demonstrates differences in predictive stability, with ATRX exhibiting the highest consistency, followed by EGFR, while IDH shows the greatest variability and TP53 displays intermediate dispersion, particularly affecting F1. (**c**) 95% confidence intervals further characterize estimate reliability, where narrower intervals for ATRX contrast with broader intervals observed for IDH, TP53, and the EGFR F1 score, indicating increased uncertainty. (**d**) Min–max ranges with superimposed mean values (black markers) illustrate the spread of performance metrics, highlighting limited dispersion for EGFR, a wider F1 range for ATRX, and comparatively greater variability for IDH and TP53, especially in F1

Cross-validation performance (Table 3; Fig. 10) reported ROC–AUCs of 0.823 ± 0.135 (95% CI: 0.688–0.958; IDH), 0.966 ± 0.020 (95% CI: 0.946–0.986; ATRX), 0.960 ± 0.029 (95% CI: 0.931–0.989; TP53), and 0.866 ± 0.024 (95% CI: 0.807–0.924; EGFR). Held-out test set AUCs were 0.971 (95% CI: 0.876–0.995) for IDH, 0.900 (95% CI: 0.717–0.989) for ATRX, 0.917 (95% CI: 0.858–0.975) for EGFR, and 0.955 (95% CI: 0.661–0.978) for TP53.

**Fig. 11:**
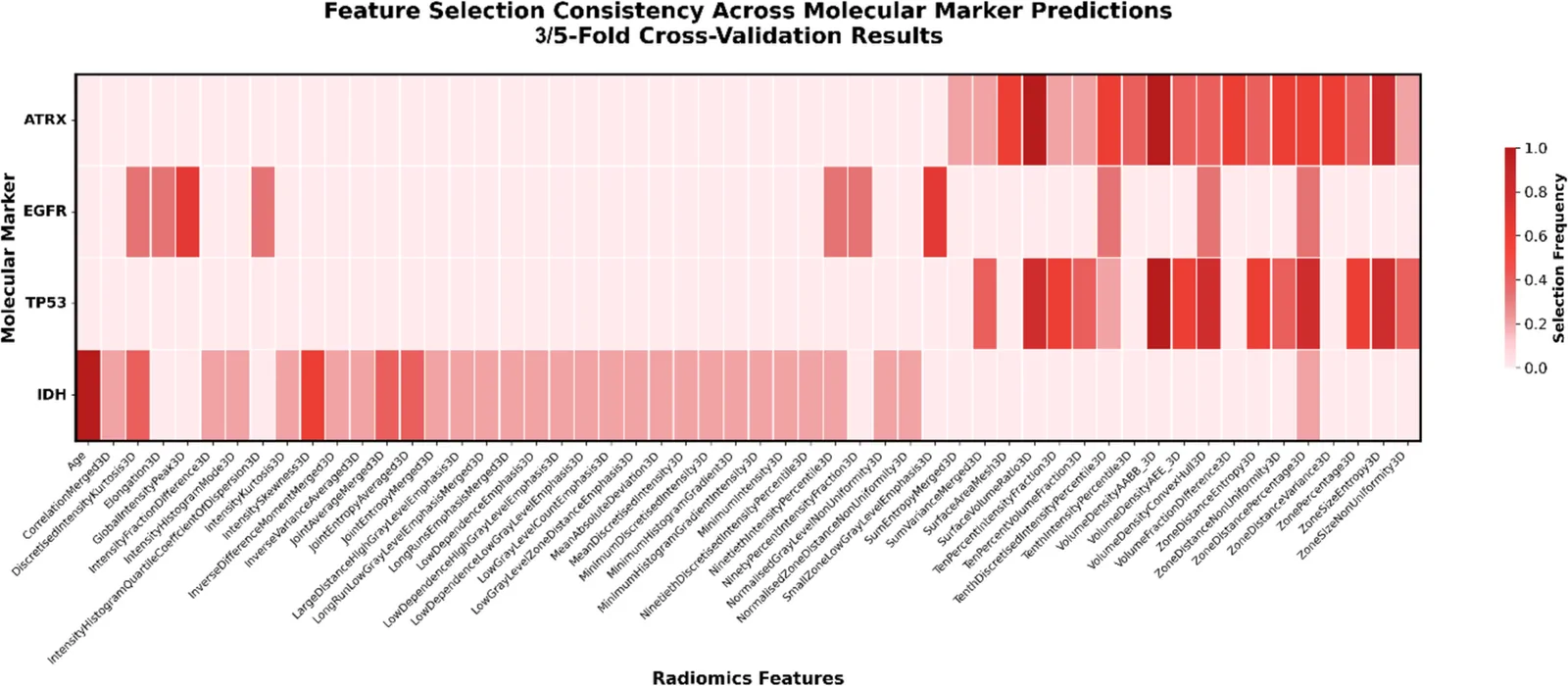
Feature-selection consistency heatmap for molecular marker prediction across cross-validation folds. The map quantifies how frequently radiomic features are selected for ATRX, TP53, and IDH (five-fold cross-validation using deep learning classifiers) and for EGFR (three-fold). Each cell represents a selection frequency ranging from 0.0 (never selected) to 1.0 (consistently selected), with a light-to-dark red gradient indicating increasing stability. Features are arranged alphabetically along the x-axis, while molecular targets are displayed on the y-axis, with gridlines delineating individual entries. Distinct, target-specific selection patterns are observed, reflecting variability in feature robustness across folds and revealing radiomic signatures characteristic of each marker. These patterns support informed feature prioritization and enhance interpretability in subsequent modelling and biological analysis

Perturbation testing resulted in average performance reductions of 0.009 for IDH and 0.010 for EGFR, corresponding to robustness scores of 0.991 and 0.990, respectively. Training AUCs were 0.977 for IDH and 0.941 for EGFR, and bootstrap analysis yielded AUCs of 0.939 ± 0.098 (95% CI: 0.867–1.000) for IDH and 0.873 ± 0.083 (95% CI: 0.620–0.986) for EGFR. Composite risk scores were 0.225 for IDH and 0.296 for EGFR. Correlation between performance metrics across model–gene combinations is summarised in Table 4, and feature selection frequency over folds is shown in Fig. 11.

**Table 3:** Cross-validation performance stability analysis across genetic mutation targets

Gene	Metric	Mean ± SD	CV (%)ᵃ	95% CI	Min–Max Range
IDH	AUC	0.823 ± 0.135	16.40	0.688–0.958	0.642–0.971
IDH	PR AUC	0.679 ± 0.124	18.33	0.555–0.803	0.521–0.825
IDH	F1 Score	0.664 ± 0.109	16.47	0.555–0.773	0.533–0.789
ATRX	AUC	0.966 ± 0.020	2.10	0.946–0.986	0.942–0.990
ATRX	PR AUC	0.994 ± 0.005	0.46	0.989–0.999	0.988–1.000
ATRX	F1 Score	0.912 ± 0.036	3.91	0.876–0.948	0.867–0.951
EGFR^b^	AUC	0.866 ± 0.024	3.25	0.807–0.924	0.833–0.889
EGFR	PR AUC	0.928 ± 0.016	2.06	0.889–0.967	0.917–0.950
EGFR	F1 Score	0.722 ± 0.039	6.65	0.624–0.820	0.667–0.750
TP53	AUC	0.960 ± 0.029	2.97	0.931–0.989	0.925–0.995
TP53	PR AUC	0.906 ± 0.074	8.15	0.832–0.980	0.815–0.987
TP53	F1 Score	0.800 ± 0.112	13.98	0.688–0.912	0.667–0.933

ᵃ CV = Coefficient of Variation (SD/Mean × 100%). CI = Confidence Interval. Results based on EGFR^b^ 3-fold, IDH, ATRX and TP53 5-fold cross-validation using top-performing models

**Table 4:** Correlation analysis between performance metrics across all model-gene combinations

Metric Pair	Correlation Coefficient	95% CI	*p*-value	Interpretation
PR AUC vs. F1 Score	*ρ* = 0.862ᵃ	0.743–0.931	< 0.001	Strong positive***
AUC vs. PR AUC	*r* = 0.457ᵇ	0.134–0.699	0.008	Moderate positive**
AUC vs. F1 Score	*r* = 0.378ᵇ	0.036–0.653	0.033	Weak positive*

ᵃ Spearman’s rank correlation coefficient (*ρ*)

ᵇ Pearson’s correlation coefficient (*r*)

Analysis based on 32 model-gene combinations. CI = Confidence Interval

* *p* < 0.05; ** *p* < 0.01; *** *p* < 0.001

## Discussion

This study demonstrates the potential of NCCT-based radiomic modelling for the non-invasive molecular characterisation of gliomas, particularly in resource-limited settings, where access to comprehensive molecular testing is often constrained and the clinical value of molecular pathological indicators requires further clarification. In such settings, even partial or estimated molecular information may retain clinical relevance by informing prognosis and supporting initial treatment decisions, despite the absence of a complete integrated diagnosis.

Across four molecular targets, the models achieved strong discrimination, with performance comparable to previously reported CT- and MRI-based studies. TabNet achieved strong discrimination for ATRX, EGFR, and TP53, with ROC–AUC values of 0.900 (95% CI: 0.717–0.989), 0.917 (95% CI: 0.858–0.975), and 0.955 (95% CI: 0.661–0.978), respectively, whereas a dense feedforward FCNN performed best for IDH prediction (AUC 0.971; 95% CI: 0.876–0.995). Collectively, these findings add to the limited literature on CT-based molecular prediction and highlight that, for selected targets, NCCT may provide diagnostically meaningful information approaching that reported for MRI, while remaining more accessible and less costly. Prior CT work on IDH classification remains limited [19]; to our knowledge, this is the first CT study to report predictive models for ATRX, EGFR, and TP53 (Tables 3 and 4; Figs. 6, 7, 8, 9 and 10).

Differences in performance across targets and algorithms likely reflect both biological variation and the way imaging features are represented. The stronger performance of TabNet for ATRX, EGFR, and TP53 suggests that these targets may depend on complex, non-linear relationships among radiomic texture and intensity features. By contrast, IDH status appears to be captured by a smaller set of dominant features, which the FCNN was able to model effectively.

Feature attribution supports this interpretation. For IDH, the model relied primarily on patient age and surface-to-volume ratio. The prominence of age is biologically plausible, as IDH mutations are more frequent in younger patients, whereas IDH-wildtype glioblastoma predominates in older adults. In a cohort enriched for glioblastoma, this association alone may provide substantial discriminative signal. This does not invalidate the model, but it does underscore the need to quantify the independent contribution of radiomic features. Future work should therefore compare age-only, radiomic-only, and combined models.

The discrepancy between internal cross-validation and held-out test performance for IDH warrants careful interpretation. Although the FCNN achieved a strong test AUC of 0.971 (95% CI: 0.876–0.995), the corresponding cross-validation estimate was lower and more variable (0.823 ± 0.135; 95% CI: 0.688–0.958). This pattern is likely attributable to the limited number of IDH-mutant cases (*n* = 26, 14.2%) and their uneven distribution across folds, which increases fold-to-fold instability and broadens uncertainty in internal validation. The higher test-set AUC may therefore partly reflect sampling variability within a small held-out subset rather than a systematic inflation of performance. In this setting, the cross-validation estimate provides the more conservative indicator of expected performance.

Across the remaining targets, feature attribution was consistent with established tumour biology. ATRX prediction relied more heavily on features reflecting intratumoural heterogeneity, in keeping with the effects of chromatin remodelling alterations [8]. EGFR prediction emphasised attenuation-related features associated with aggressive tumour phenotypes [6], whereas TP53 prediction drew on a broader feature set, consistent with its diverse biological role [5].

A central issue in glioma radiogenomics is the overlap between tumour grade and molecular status. Imaging features such as necrosis, oedema, mass effect, and heterogeneous peripheral structure are common in high-grade disease, particularly IDH-wildtype glioblastoma. In a cohort dominated by glioblastoma (78.2%), models may therefore appear to predict molecular status while primarily capturing high-grade imaging patterns. This may partly account for the performance of the IDH and ATRX models, which may reflect discrimination between GBM-like and non-GBM imaging phenotypes rather than purely molecular differences.

EGFR evaluation is further constrained by non-uniform molecular testing and the small number of evaluated cases. This resulted in class imbalance and limited statistical power. The enrichment of glioblastoma within the EGFR-tested subgroup also introduces selection bias that should be considered when interpreting these results. Although resampling was applied within the training data and sensitivity checks were performed, the resulting estimates remain uncertain. More systematic EGFR testing across the full cohort, including lower-grade gliomas, is needed to better define EGFR-specific imaging signatures.

Robustness experiments were conducted to evaluate model stability in cohorts with limited sample size and variable performance for certain molecular targets. The tests showed that the models were relatively stable under simulated perturbation, with minimal performance degradation, as reflected by robustness scores of 0.991 for IDH and 0.990 for EGFR. Composite risk scores were similarly low (0.225 for IDH and 0.296 for EGFR), supporting consistent model behaviour across resampling, training, and perturbation-based evaluations. In practical terms, this denotes that predictive behaviour remained relatively stable across validation procedures and implies resilience to modest variation in radiomic feature computation arising from scanner-related noise or minor segmentation differences. These findings do not establish external validity, however, and should not be interpreted as evidence of generalisability beyond the present study setting.

The distribution of molecular alterations reflects both the glioblastoma-dominant composition of the cohort and the selective application of molecular testing. The high prevalence of ATRX loss and the low rate of IDH mutation are consistent with GBM predominance, but subgroup-based testing limits representativeness. This should be considered when interpreting both prevalence and model performance, particularly with respect to broader glioma populations.

From a clinical perspective, CT-based molecular prediction is most relevant in settings where access to advanced imaging or molecular testing is constrained. In such contexts, NCCT-derived estimates may provide early, non-invasive information that can assist in preliminary risk stratification and guide initial management decisions when full molecular profiling is not available. These methods are not a substitute for histopathological diagnosis, but they may add value during initial assessment, especially when tissue results are delayed.

NCCT remains inherently limited for soft-tissue characterisation. The absence of contrast limits the indirect assessment of blood–brain barrier disruption., and its lower soft-tissue contrast relative to MRI limits comprehensive delineation of tumour extent and microenvironment. Nevertheless, the present findings indicate that clinically relevant information can still be derived from NCCT, supporting its complementary role in emergency and resource-constrained settings when MRI is unavailable or delayed. Accordingly, these results should be interpreted as evidence of adjunctive value and should not be considered an alternative to MRI-based radiogenomic assessment.

The marked predominance of glioblastoma restricts the exploration of WHO 2021 glioma subtyping, which is defined primarily by IDH mutation status and 1p/19q co-deletion. With glioblastoma accounting for nearly 80% of the cohort, the dataset is markedly imbalanced and does not permit robust CT-based discrimination among major molecular subtypes. As a result, the present models are more likely to reflect GBM-associated imaging patterns than to achieve true molecular subtype classification, representing an important shortcoming of the study design.

This study is limited by its retrospective single-centre design and lack of external validation. Model performance may have been influenced by institution-specific imaging protocols. The EGFR model is especially affected by the small number of positive cases, and class imbalance across targets contributes to statistical uncertainty. Additional limitations include single-reader segmentation without inter-reader reproducibility assessment and the absence of decision curve analysis, which restricts evaluation of clinical utility.

Future work should focus on multicentre validation in larger and more representative cohorts. Multi-reader segmentation with reproducibility assessment would help quantify annotation variability. Methodological refinements, including ensemble modelling and integration with MRI where available, may further improve performance and robustness.

## Conclusion

Radiogenomic approaches using NCCT can provide additional information for the non-invasive molecular evaluation of gliomas. Although histopathological and molecular analyses remain the reference standard, CT-based estimates may still offer early indications during the initial diagnostic workup. In this study, the models predicted key glioma-associated genetic alterations with performance comparable to established reference methods, indicating potential value for preoperative decision-making in resource-constrained or emergency settings.

However, these findings should be interpreted as exploratory rather than practice-changing. The cohort was predominantly enriched for glioblastoma, the study lacked external validation, and the confounding effect of tumour grade on molecular signals has not been fully resolved. Larger, multicentre prospective studies with externally validated cohorts are needed before these models can be considered for routine clinical use. The EGFR-related results, based on a very small sample, remain particularly preliminary and require replication across multiple scanner platforms and patient populations.

## Supplementary Information

Below is the link to the electronic supplementary material.

Supplementary Material 1 (DOCX 36.0 KB)

Supplementary Material 2 (DOCX 788 KB)

## Data Availability

The data used in this study were collected at Dokuz Eylul University Hospital and are currently not publicly available due to ongoing research activities.
