# Supplementary material for: CT-based deep learning radiogenomics for predicting key glioma genotypes (IDH, ATRX, EGFR, TP53)
Source: Neuroradiology. 2026 Jun 4;68(9):2859–75. doi: 10.1007/s00234-026-04043-3 (PMC13680328; doi:10.1007/s00234-026-04043-3)
Supplement: Supplementary file 1 [file 234_2026_4043_MOESM1_ESM.docx]

**Appendix**

**Statistical Analysis: Theoretical Background**

*Supplement to the Methods Section — Statistical Analysis*

# A1. Overview

This appendix provides the theoretical foundation for the statistical procedures described in the Methods section. Three interconnected methodological components are covered: (i) the selection and computation of binary classification performance metrics; (ii) the Bias-Corrected and Accelerated (BCa) bootstrap procedure for confidence interval estimation on the held-out test set; and (iii) the corrected resampled *t*-statistic for confidence interval estimation across cross-validation folds. Together, these components constitute a statistically rigorous, reproducible framework for model evaluation in small-sample radiomics settings.

# A2. Performance Metrics

## A2.1 Rationale for Metric Selection

Model performance was characterised using a complementary set of fifteen metrics, each capturing a distinct aspect of discriminative and calibrative ability. ROC-AUC quantifies rank-order discrimination across all possible decision thresholds and is robust to class imbalance at the population level. PR-AUC is the preferred summary metric for imbalanced datasets because it weights performance on the minority class more heavily, being equivalent to the average precision weighted by recall at each threshold. F1 Score provides a threshold-specific harmonic mean of precision and recall and is sensitive to both false-positive and false-negative errors simultaneously.

Sensitivity and Specificity decompose the confusion matrix into clinically interpretable components — the true-positive rate and true-negative rate respectively — and are the standard reporting metrics in diagnostic medicine. Their ratio yields the Positive and Negative Likelihood Ratios (LR+, LR−), which express how much a test result shifts the pre-test probability of disease and are directly applicable at the patient level. The Diagnostic Odds Ratio (DOR = LR+ / LR−) provides a single summary of discriminative power on the odds scale.

Matthews Correlation Coefficient (MCC) is widely regarded as the most informative single-number classifier metric for binary classification, as it accounts for all four cells of the confusion matrix and is invariant to class imbalance. Balanced Accuracy is the arithmetic mean of sensitivity and specificity, providing an imbalance-adjusted accuracy estimate. The Brier Score is a proper scoring rule measuring the mean squared difference between predicted probabilities and observed outcomes, simultaneously assessing calibration and sharpness.

Net Reclassification Improvement (NRI) and Integrated Discrimination Improvement (IDI) are reclassification metrics that quantify the added predictive value of the proposed model relative to a logistic regression baseline trained on the same feature set. The category-free (continuous) NRI variant was used, as it does not require pre-specified risk categories and is appropriate for continuous probability outputs.

## A2.2 Decision Threshold

A fixed classification threshold of τ = 0.5 was applied to convert predicted probabilities into binary class assignments for threshold-dependent metrics (F1, Sensitivity, Specificity, PPV, NPV, MCC, Balanced Accuracy, LR+, LR−, DOR). Metrics operating on continuous probability outputs (ROC-AUC, PR-AUC, Brier Score, NRI, IDI) were computed directly from the raw model probabilities without thresholding.

*Table A1. Performance metrics, computational formulae, input types, and CI methods applied.*

| **Metric** | **Formula / Function** | **Input type** | **CI method applied** |
| --- | --- | --- | --- |
| ROC-AUC | *roc_auc_score(y, ŷ_prob)* | Probability | Both |
| PR-AUC | *average_precision_score(y, ŷ_prob)* | Probability | Both |
| F1 Score | *f1_score(y, ŷ_pred)* | Binary | Both |
| Sensitivity | *TP / (TP + FN)* | Binary | Test set |
| Specificity | *TN / (TN + FP)* | Binary | Test set |
| PPV | *TP / (TP + FP)* | Binary | Test set |
| NPV | *TN / (TN + FN)* | Binary | Test set |
| MCC | *matthews_corrcoef(y, ŷ_pred)* | Binary | Test set |
| Balanced Accuracy | *balanced_accuracy_score(y, ŷ_pred)* | Binary | Test set |
| Brier Score | *brier_score_loss(y, ŷ_prob)* | Probability | Test set |
| LR+ | *Sensitivity / (1 − Specificity)* | Binary | Test set |
| LR− | *(1 − Sensitivity) / Specificity* | Binary | Test set |
| DOR | *LR+ / LR−* | Binary | Test set |
| NRI | *NRI_events + NRI_non-events* | Probability | Test set |
| IDI | *IDI_events + IDI_non-events* | Probability | Test set |

**Note:** *Binary inputs use τ = 0.5 decision threshold. ŷ_prob = predicted probability; ŷ_pred = binary prediction. TP = true positive; TN = true negative; FP = false positive; FN = false negative.*

# A3. Pairwise ROC-AUC Comparison: DeLong's Test

Pairwise comparisons between ROC curves were conducted using DeLong's non-parametric test, which frames the AUC as a Mann-Whitney U statistic and derives its variance analytically via the structural component approach, avoiding bootstrap resampling entirely. Specifically, for two correlated AUC estimates (*e.g.*, two classifiers evaluated on the same test set), the covariance matrix of the AUC pair is estimated from the placement values of the positive and negative samples, yielding a *χ*² test statistic with 1 degree of freedom. This approach is statistically equivalent to the Wilcoxon signed-rank test for correlated AUCs and is the recommended method for head-to-head ROC comparison in paired experimental designs. All pairwise comparisons were two-tailed with significance set at p < 0.05.

# A4. BCa Bootstrap Confidence Intervals (Held-Out Test Set)

## A4.1 Motivation

Radiomics cohorts are characteristically small (typically n < 200), and the sampling distributions of metrics such as AUC, MCC, F1 Score, and Brier Score are markedly non-normal in small samples — particularly when point estimates approach the boundaries of their defined ranges. Standard asymptotic (Wald-type) confidence intervals assume normality of the sampling distribution and are unreliable in these regimes, producing intervals with empirically poor coverage probabilities. The BCa bootstrap was therefore adopted as the primary CI estimation method for all metrics derived from the held-out test set, as it is non-parametric, requires no distributional assumptions, and achieves second-order accuracy through two analytic corrections described below.

## A4.2 Bootstrap Sampling Distribution

For a given metric function *f*(·), B = 2,000 bootstrap resamples {(y**ᵢ*, ŷ**ᵢ*)}ᵦ were drawn with replacement from the n observations of the held-out test set. For each resample, the metric was computed:

**θ̂*(b) = f( y*(b), ŷ*(b) ), b = 1, 2, …, B**	*(Eq. A1)*

yielding the empirical bootstrap distribution {θ̂*(1), …, θ̂*(B)}. A conservative fallback was applied in rare cases where a resample contained only a single class (assigning θ̂*(b) = θ̂), preventing undefined metric computations under complete class separation.

## A4.3 Bias-Correction Factor (z₀)

The observed bootstrap distribution median frequently diverges from the point estimate θ̂, especially for bounded metrics. The BCa method corrects for this via the bias-correction factor z₀, defined as the probit of the proportion of bootstrap replicates falling below the observed statistic:

**z₀ = Φ⁻¹( #{b : θ̂*(b) < θ̂} / B )**	*(Eq. A2)*

where Φ⁻¹ denotes the inverse standard normal cumulative distribution function (probit function). When the bootstrap distribution is symmetric and unbiased, z₀ = 0 and no correction is applied.

## A4.4 Acceleration Factor (â)

The acceleration factor â corrects for the rate of change of the standard error of θ̂ with respect to the true parameter value — a phenomenon producing asymmetric confidence intervals near the extremes of bounded metrics. It is estimated via the jackknife influence function:

**â = Σᵢ (θ̄₋ᵢ − θ̂₋ᵢ)³ / 6 · [ Σᵢ (θ̄₋ᵢ − θ̂₋ᵢ)² ]^(3/2)**	*(Eq. A3)*

where θ̂₋ᵢ is the metric computed with the *i*-th observation omitted and θ̄₋ᵢ is the mean of all n jackknife estimates. When â = 0, the BCa interval reduces to the standard bias-corrected (BC) percentile interval.

## A4.5 Adjusted Percentile Endpoints

The BCa 95% confidence interval is constructed by adjusting the standard percentile endpoints using both z₀ and â. The corrected lower (p_lo) and upper (p_hi) quantile positions are:

**p_lo = Φ( z₀ + (z₀ + z_{α/2}) / (1 − â(z₀ + z_{α/2})) )**	*(Eq. A4)*

**p_hi = Φ( z₀ + (z₀ + z_{1−α/2}) / (1 − â(z₀ + z_{1−α/2})) )**	*(Eq. A5)*

where z_{α/2} = Φ⁻¹(0.025) ≈ −1.96 and z_{1−α/2} = Φ⁻¹(0.975) ≈ +1.96 for a two-sided 95% CI. Both p_lo and p_hi are clipped to [0, 1]. The BCa interval is then the corresponding empirical percentiles of the bootstrap distribution:

**95% CI = [ θ̂*(p_lo · 100), θ̂*(p_hi · 100) ]**	*(Eq. A6)*

## A4.6 Statistical Properties

The BCa bootstrap achieves second-order accuracy — meaning the coverage error converges at rate O(n⁻¹) as opposed to O(n⁻¹/²) for the naïve percentile bootstrap — and is transformation-invariant, a property of particular importance for bounded metrics such as AUC and Brier Score whose sampling distributions are inherently asymmetric. These properties have been formally established by Efron (1987) and DiCiccio & Efron (1996) and recommended for small-sample clinical prediction modelling by Steyerberg et al. (2010).

# A5. Cross-Validation Confidence Intervals: Corrected Resampled t-Statistic

## A5.1 The Problem of Fold Dependence

When k-fold cross-validation is used to estimate model performance, the naïve confidence interval formula — mean ± 1.96 × (SD / √k) — is statistically invalid because it incorrectly assumes statistical independence between folds. In k-fold CV, training sets of adjacent folds share (k−2)/(k−1) of their observations, inducing positive correlation between per-fold metric estimates. Treating these estimates as independent underestimates the true variance of the mean, producing confidence intervals that are systematically too narrow and inflating Type I error rates in model comparison.

## A5.2 Corrected Variance Estimator

Nadeau & Bengio (2003) demonstrated that the standard variance estimator σ²/k must be scaled by a correction factor that accounts for the degree of overlap between training folds. The corrected variance estimate is:

**σ²_corr = ( 1/k + n₂/n₁ ) · σ²**	*(Eq. A7)*

where k is the number of folds, n₁ is the training fold size, n₂ is the validation fold size, and σ² = (1/(k−1)) Σⱼ (xⱼ − x̄)² is the sample variance of the per-fold metric estimates across the k folds. The term n₂/n₁ approximates the correlation between fold estimates arising from training-set overlap, and is the key correction that distinguishes this estimator from the naïve formula.

## A5.3 Confidence Interval Construction

The 95% confidence interval for the cross-validation performance estimate is:

**95% CI = x̄ ± t_{k−1, α/2} · √σ²_corr**	*(Eq. A8)*

where x̄ is the mean metric across k folds and t_{k−1, α/2} is the α/2 quantile of Student's t-distribution with k−1 degrees of freedom. For k = 5 and α = 0.05, t_{4, 0.025} = 2.776, which is wider than the normal quantile z_{0.025} = 1.96, appropriately reflecting the increased uncertainty from a small number of folds.

# A6. Feature Importance Methods

## A6.1 TabNet Attention Weights

TabNet employs a sequential attention mechanism that learns sparse, instance-level feature masks at each decision step. At each step *i*, a softmax-normalised attention mask h*i*(x) is applied to the input features, enforcing sparsity via an entropy regularisation term. Aggregate feature importance is derived by averaging the attention masks across all decision steps and all training instances, yielding a global importance score for each input feature. This mechanism provides inherent interpretability without requiring post-hoc attribution methods.

## A6.2 Permutation Importance

For tree-based classifiers, feature importance was assessed using the permutation importance method, which directly measures the decrease in model performance when the values of a single feature are randomly shuffled, breaking its association with the target variable. Formally, the importance of feature *j* is defined as:

**I_j = Performance(M) − E[ Performance(M_π_j) ]**	*(Eq. A9)*

where M denotes the fitted model evaluated on the test set, and M_π_j denotes evaluation after randomly permuting column j. Permutation importance is model-agnostic and directly tied to held-out performance, making it more reliable than impurity-based (Gini) importance for correlated or high-dimensional feature sets.

# A7. Summary of Mathematical Notation

*Table A2. Mathematical notation used throughout the statistical analysis.*

| **Symbol** | **Definition** |
| --- | --- |
| *θ̂* | Observed point estimate of the performance metric |
| *θ̂*(b)* | Metric value computed on the b-th bootstrap resample |
| *B* | Total number of bootstrap resamples (B = 2,000) |
| *α* | Significance level (α = 0.05) |
| *z₀* | Bias-correction factor |
| *â* | Acceleration factor (jackknife-derived) |
| *θ̂₋ᵢ* | Jackknife leave-one-out metric estimate (i-th obs. removed) |
| *θ̄₋ᵢ* | Mean of all jackknife leave-one-out estimates |
| *Φ⁻¹( · )* | Probit function (inverse of standard normal CDF) |
| *p_lo, p_hi* | BCa-adjusted lower and upper percentile endpoints |
| *k* | Number of cross-validation folds (k = 5, k = 3) |
| *n₁* | Training fold size |
| *n₂* | Validation fold size |
| *x̄* | Mean metric across k folds |
| *σ²* | Variance of the metric across k folds |
| *σ²_corr* | Corrected variance estimate (Nadeau & Bengio, 2003) |

# A8. References

**[1]** Efron B. Better bootstrap confidence intervals. J Am Stat Assoc. 1987;82:171–185. <https://doi.org/10.1080/01621459.1987.10478410>

**[2]** DiCiccio TJ, Efron B. Bootstrap confidence intervals. Stat Sci. 1996;11:189–228. <https://doi.org/10.1214/ss/1032280214>

**[3]** Nadeau C, Bengio Y. Inference for the generalization error. Mach Learn. 2003;52:239–281. https://doi.org/10.1023/A:1024068626366

**[4]**	DeLong ER, DeLong DM, Clarke-Pearson DL. Comparing the areas under two or more correlated receiver operating characteristic curves: a nonparametric approach. Biometrics. 1988;44:837–845. https://doi.org/10.2307/2531595

**[5]**	Arik SO, Pfister T. TabNet: attentive interpretable tabular learning. Proc AAAI Conf Artif Intell. 2021;35:6679–6687. https://doi.org/10.1609/aaai.v35i8.16826

**[6]** Friedman JH. Greedy function approximation: a gradient boosting machine. Ann Stat. 2001;29:1189–1232**.** <https://doi.org/10.1214/aos/1013203451>

**[7]**	Breiman L. Random forests. Mach Learn. 2001;45:5–32. https://doi.org/10.1023/A:1010933404324
