# Supplementary material for: CT-based deep learning radiogenomics for predicting key glioma genotypes (IDH, ATRX, EGFR, TP53)
Source: Neuroradiology. 2026 Jun 4;68(9):2859–75. doi: 10.1007/s00234-026-04043-3 (PMC13680328; doi:10.1007/s00234-026-04043-3)
Supplement: Supplementary file 2 [file 234_2026_4043_MOESM2_ESM.docx]

**Supplementary Figures**

**Fig. S1** Classification flowchart of adult-type diffuse gliomas according to WHO 2021 criteria.

**Supplementary Tables**

**Table S1.** Hyperparameter configurations and optimisation settings for machine learning models in the study (n = 6 models).

| Algorithm | Key Parameters | Hyperparameter Range/Value |
| --- | --- | --- |
| k-Nearest Neighbors (k-NN) | n_neighbors=5 weights='uniform' | n_neighbors: [1–30] weights: ['uniform', 'distance'] |
| Logistic Regression (LR) | C=1.0 penalty='l2' | C: [$1\times10⁻⁴$–$1\times10⁴$] (log) solver: ['lbfgs'a, 'liblinear'b] |
| Multilayer Perceptron (MLP) | hidden_layer_sizes=(100,) early_stopping=True | hidden_layer_sizes: [(50,), (100,), (100,50)] alpha: [$1\times10⁻⁵$–$1\times10⁻¹$] (log) |
| Random Forest (RF) | n_estimators=100 | n_estimators: [50–500] max_depth: [3–15] |
| Support Vector Machine (SVM) | kernel='rbf'c probability=True | C: [$1\times10⁻^{2}$–$1\times10^{3}$] (log) gamma: [$1\times10⁻⁴$–$10$] (log) |
| XGBoost (XGB) | eval_metric='logloss' | max_depth: [3–10] learning_rate: [0.01–0.3] |

**Footnotes:**
^a^ Limited-memory Broyden–Fletcher–Goldfarb–Shanno

^b^ library for large linear classification
^c^ Radial Basis Function kernel

^d^ All models used stratified 5-fold cross-validation and Tree-structured Parzen Estimator optimisation (50 trials).
^e^ Parameters sourced from scikit-learn (v1.3) and XGBoost (v1.7) documentation.
^f^ Log-scale indicates logarithmic sampling during hyperparameter search.

**Table S2.** Performance metrics of machine learning models for predicting genetic alterations in gliomas.

| Gene | Model | AUC | F1 Score | PR AUC | Composite Score | Composite Rank |
| --- | --- | --- | --- | --- | --- | --- |
| ATRX | **TabNet** | **0.900** | **0.926** | **0.983** | **0.936** | **1** |
|  | Random Forest | 0.739 | 0.793 | 0.952 | 0.828 | 2 |
|  | Fully connected neural network | 0.691 | 0.786 | 0.938 | 0.805 | 3 |
|  | SVM | 0.671 | 0.918 | 0.932 | 0.840 | 4 |
|  | K-Nearest Neighbors | 0.593 | 0.750 | 0.871 | 0.738 | 5 |
|  | MLP | 0.586 | 0.745 | 0.903 | 0.745 | 6 |
|  | Logistic Regression | 0.579 | 0.735 | 0.903 | 0.739 | 7 |
|  | XGBoost | 0.525 | 0.793 | 0.880 | 0.733 | 8 |
| EGFR | **TabNet** | **0.917** | **0.889** | **0.950** | **0.919** | **1** |
|  | Random Forest | 0.750 | 0.667 | 0.854 | 0.757 | 2 |
|  | SVM | 0.750 | 0.667 | 0.893 | 0.770 | 3 |
|  | K-Nearest Neighbors | 0.750 | 0.000 | 0.768 | 0.506 | 4 |
|  | Fully connected neural network | 0.667 | 0.750 | 0.830 | 0.749 | 5 |
|  | Logistic Regression | 0.583 | 0.667 | 0.793 | 0.681 | 6 |
|  | MLP | 0.583 | 0.571 | 0.793 | 0.649 | 7 |
|  | XGBoost | 0.458 | 0.333 | 0.554 | 0.448 | 8 |
| IDH | **Fully connected neural network** | **0.971** | **0.737** | **0.904** | **0.871** | **1** |
|  | Logistic Regression | 0.967 | 0.667 | 0.886 | 0.840 | 2 |
|  | K-Nearest Neighbors | 0.929 | 0.583 | 0.640 | 0.717 | 3 |
|  | Random Forest | 0.924 | 0.625 | 0.651 | 0.733 | 4 |
|  | MLP | 0.914 | 0.600 | 0.795 | 0.770 | 5 |
|  | XGBoost | 0.910 | 0.625 | 0.620 | 0.718 | 6 |
|  | SVM | 0.900 | 0.571 | 0.789 | 0.753 | 7 |
|  | TabNet | 0.891 | 0.750 | 0.754 | 0.798 | 8 |
| TP53 | **TabNet** | **0.955** | **0.727** | **0.917** | **0.866** | **1** |
|  | Fully connected neural network | 0.659 | 0.500 | 0.658 | 0.606 | 2 |
|  | SVM | 0.591 | 0.421 | 0.392 | 0.468 | 3 |
|  | MLP | 0.568 | 0.333 | 0.505 | 0.469 | 4 |
|  | Logistic Regression | 0.545 | 0.000 | 0.487 | 0.344 | 5 |
|  | K-Nearest Neighbors | 0.511 | 0.500 | 0.463 | 0.491 | 6 |
|  | XGBoost | 0.432 | 0.000 | 0.282 | 0.238 | 7 |
|  | Random Forest | 0.261 | 0.000 | 0.221 | 0.161 | 8 |

**Footnotes:**
• Bold entries indicate top-performing models for each gene.
• AUC = Area Under the ROC Curve; PR AUC = Precision-Recall Area Under Curve.
• Composite Score = Weighted average of AUC, F1, and PR AUC.
